# Ameliorative effects of humic acid and L‐tryptophan on enzyme activity, mineral content, biochemical properties, and plant growth of spinach cultivated in saline conditions

**DOI:** 10.1002/fsn3.4435

**Published:** 2024-08-30

**Authors:** Nezahat Turfan, Beyhan Kibar, Nazakat Davletova, Hakan Kibar

**Affiliations:** ^1^ Department of Biology, Faculty of Science Kastamonu University Kastamonu Türkiye; ^2^ Department of Horticulture, Faculty of Agriculture Bolu Abant Izzet Baysal University Bolu Türkiye; ^3^ Department of Seed Science and Technology, Faculty of Agriculture Bolu Abant Izzet Baysal University Bolu Türkiye

**Keywords:** amino acids, biochemical composition, foliar application, humic substances, *Spinacia oleracea* L.

## Abstract

Salinity poses a significant abiotic stress that limits plant productivity, thereby posing a serious threat to agricultural sustainability and worldwide food security. Techniques that can overcome this problem are needed. Recent focus has been placed on employing organic substances like humic acid (HA) and amino acids, including L‐tryptophan (L‐TRP), to mitigate the negative effects of salt stress on cultivated plants. Accordingly, in this research, the impact of foliar applications of HA and L‐TRP, both separately and combined, on the growth parameters and biochemical properties of spinach subjected to salt stress was investigated. In the present study, eight treatments (1. control, 2. salt (NaCl), 3. HA, 4. L‐TRP, 5. HA + NaCl, 6. L‐TRP + NaCl, 7. HA + L‐TRP, and 8. HA + L‐TRP + NaCl) were investigated. The study showed that salt stress markedly reduced several growth properties in spinach, including plant height, number of leaves, leaf dimensions, and both fresh and dry weight. Additionally, it significantly lowered contents of chlorophyll (*a*, *b*, and total), carotenoid, polyphenol, lutein, anthocyanin, polyphenol oxidase, glycine betaine, relative water content, and the antioxidant enzyme activities (ascorbate peroxidase, catalase, peroxidase, and superoxide dismutase). On the other hand, significant increases were observed in sodium, chlorine, potassium, sulfur, zinc, nickel, proline, malondialdehyde, and hydrogen peroxide levels of spinach with salinity. Individual and combined applications of HA and L‐TRP positively influenced plant growth, relative water content, activities of antioxidant enzyme, chlorophyll, and mineral contents of spinach under both normal and saline conditions. In conclusion, the combined use of HA and L‐TRP under salt stress conditions is promising in mitigating the negative impacts of salinity and can be suggested as an effective alternative approach for cultivating spinach in saline environments.

## Graphical abstract

Nowadays, soil salinity is considered a major environmental threat in terms of the future of plant production in many fertile regions of the world and Türkiye. Results of the present study indicated that plant growth, enzyme activity, mineral content, and plant growth of spinach were significantly affected by salt stress, humic acid, and L‐tryptophan. It has been determined that humic acid and L‐tryptophan treatments play a significant role in reducing the negative effects of salt stress.

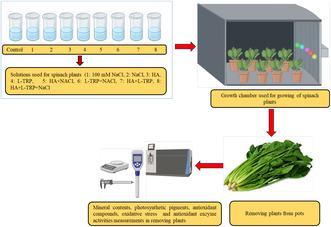

## 1 INTRODUCTION

Spinach (*Spinacia oleracea* L.) is a vegetable of significant economic value and is a member of the Amaranthaceae family (Caparrotta et al., 2019). It is an annual cool climate vegetable grown worldwide for its leaves. The homeland of spinach is southwestern and central Asia. Spinach has a significant place in human nutrition because of its rich variety of vitamins and minerals (Nishihara et al., 2001). It comes to the forefront, especially with its high Fe content, vitamin A, B2, and C contents, and low calories. Spinach also contains large quantities of bioactive compounds (Pandjaitan et al., 2005). Spinach has a short vegetation period. Spinach is widely grown and consumed in Türkiye as well as in the world. In 2022, Türkiye ranked third in the world with a spinach production of 230,071 tons (FAO, 2024). Salinity is one of the key stress factors that significantly limits spinach production. Spinach is moderately sensitive to salt (Ferreira et al., 2020). The tolerance threshold of spinach was reported as 2 dS m^−1^ (Shannon & Grieve, 1998). Salt stress reduce germination, seedling growth, root elongation, chlorophyll content, and photosynthesis in spinach (Kaya et al., 2002).

Salinity stress is a significant abiotic factor that impairs plant growth and yields globally (Balasubramaniam et al., 2023). It severely limits crop production globally. Soil salinity is prevalent in many arid and semiarid areas globally, characterized by low rainfall and high evaporation rates (Ibekwe et al., 2017). Roughly 20% of global agricultural land suffers from salinity issues. An estimated 45 million hectares of irrigated land, which produce one‐third of the world's food supply, suffer from salt damage (Machado & Serralheiro, 2017). Soil salinity directly harms numerous agriculturally significant plant species, leading to substantial crop losses globally each year. A plant's response to salinity is influenced by its genotype, the stage of growth, and the characteristics and severity of the salinity (Ventura et al., 2014). Among various salinity types, stress caused by NaCl is considered the most extreme. Soil salinity modifies the soil's physical and chemical properties, resulting in soil structure degradation and a reduction in agriculturally viable land. Salt stress adversely impacts various physiological and metabolic processes, including lipid metabolism, photosynthesis, respiration, protein synthesis, and nutrient absorption in plants. Consequently, this stress can drastically diminish plant growth, yield, and quality (Parida & Das, 2005). Moreover, increased salinity levels can cause a significant increase in the production of reactive oxygen species (ROS), which may lead to oxidative damage to various cellular components (Ventura et al., 2014).

Türkiye is among the nations grappling with severe salinity issues. Soil salinity is an increasing agricultural problem in our country. In Türkiye, salinity affects 1.5 million hectares of land (Okur & Örçen, 2020). Agricultural sustainability is threatened by soil salinity. For this reason, it is imperative to determine appropriate strategies to alleviate the detrimental effects of salt stress on agriculturally important plants, both within the nation and worldwide.

The recent focus on developing methods and strategies for the adverse effects of salinity on crops and enhancing plant salinity tolerance has attracted considerable attention. The use of organic substances like humic acid and amino acids like L‐tryptophan has been emphasized to counteract the negative impact of soil salinity on plant growth (Akladious & Mohamed, 2018; Hanci, 2019).

The main components of soil organic matter are humic substances, which make up 65–70% of the total. These substances enhance soil fertility, promote the growth of microorganisms, improve nutrient availability, and thus have a positive impact on plant growth and productivity. Furthermore, humic substances alleviate the negative impacts of salinity and contribute to the maintenance of sustainable agriculture (Canellas et al., 2015). They exhibit antioxidant properties that suppress the production of ROS and protect cells from oxidative harm (Al‐Falahi et al., 2022). Humic substances are divided into three categories: humin, humic acid (HA), and fulvic acid. HA is a vital component of humic substances and is nutrient rich, as reported by Canellas et al. (2015). According to Bacilio et al. (2016), HA improves soil properties, increases nutrient availability, and enhances plants' metabolic functions. HA has a positive impact on plant growth and development, both directly and indirectly. It impacts photosynthesis, enzyme activity, protein synthesis, and respiration, as well as water and nutrient absorption, thereby increasing crop yields, as stated by Arjumend et al. (2015). Furthermore, HA enhances plant tolerance to diverse environmental stresses. HA significantly improves nutrient absorption in plants, particularly in saline soils, mitigating the detrimental impacts of salt stress. Studies have shown that HA activates the plants' enzymatic defense against stress (Çimrin et al., 2010). Additionally, HA minimizes membrane damage, which may promote salt tolerance (Canellas et al., 2015). HA is regarded as a potential solution for mitigating the negative impacts of salt stress. Past studies indicate that the exogenous treatment of HA enhances plant growth and strengthens the plant's resistance to salt stress, as documented in studies by Kalyoncu et al. (2017), Akladious and Mohamed (2018), Naseri et al. (2021), Al‐Falahi et al. (2022), and Gabr et al. (2022).

Recent studies are focusing more on the role of amino acids in enhancing plant tolerance to salinity. L‐tryptophan (L‐TRP) is an essential amino acid for humans, plants, animals, and certain bacteria (Frankenberger & Arshad, 1991). In plants, it acts as a precursor to indole acetic acid (IAA), melatonin, and serotonin. The significance of L‐TRP in auxin synthesis underscores its crucial role. Its functional roles in germination and plant growth are well documented (Hanci, 2019). Exogenous treatment of L‐TRP increases auxin levels in plant tissues. L‐TRP serves as an osmolyte, modulates ion transport, assists in stomatal opening, and mitigates the adverse effects of heavy metals (Rai, 2002). Additionally, it enhances plant resilience against abiotic stress factors (Alfosea‐Simón et al., 2020; Hussein et al., 2014; Jamil et al., 2018). L‐TRP can be administered to plants through different methods including soil application, foliar spraying, and seed treatment. L‐TRP has been investigated in previous studies to cope with and overcome salt stress in different vegetables (Hanci, 2019; Hussein et al., 2014; Jamil et al., 2018).

To our knowledge, research on the impact of exogenous HA and L‐TRP on spinach's salinity tolerance is scarce. We hypothesized that both separate and combined treatments of HA and L‐TRP might enhance the growth and salt stress resilience of spinach. As a result, the objective of this study was to investigate the effects of foliar applications of HA and L‐TRP, individually and in combination, on the growth parameters, biochemical composition, antioxidant compounds, and oxidative stress of spinach cultivated in saline environments.

## 2 MATERIALS AND METHODS

### 2.1 Material

In the present study, spinach seeds (*Spinacia oleracea* L. cv. Efsun F1) were used as plant material. Spinach seeds were purchased from the MAY Seed Company (Bursa, Türkiye). Salt (NaCl) and L‐tryptophan used in the study were obtained from Merck (Darmstadt, Germany). Humic acid (HA) was purchased from Humate Chemistry (Humata‐12, Kocaeli, Türkiye). HA used in the study had 10% total organic matter, 12% total humic acid + fulvic acid, 1.5% water‐soluble K_2_O, and 10.5–12.5 pH content.

### 2.2 Method

#### 2.2.1 Salt, humic acid, and L‐tryptophan treatments

A total of eight different treatments, consisting of individual and combined uses of salt, HA, and L‐TRP, were examined in the study (Table 1). No salt, HA, or L‐TRP was added to the control treatment. The doses of salt, HA, and L‐TRP were selected based on preliminary experiments. To determine the toxic dose of salt, an experiment was carried out with seeds in petri dishes. Seeds were soaked in salt solutions prepared at different concentrations (0, 50, 75, 100, 150, 200, and 250 mM) for 2 h and then planted in petri dishes containing filter paper. The concentration that reduced germination by more than 60% compared to the control was chosen as the toxic dose. To detect the stimulating dose of L‐TRP and HA, the seeds were soaked in L‐TRP (0, 1.2, 2.4, 3.8, and 5.0 mM) and HA (0, 5, 10, 15, 20, and 25 mL/L) solutions prepared at different concentrations for 2 h and planted in petri dishes containing filter paper. Doses that stimulated germination by more than 60% compared to the control were selected. As a result, 200 mM dose of NaCl, 3.8 mM dose of L‐TRP, and 25 mL/L dose of HA were used.

**TABLE 1:** Treatments in research and their abbreviations.

Treatment number	Content	Abbreviation
1	No salt, humic acid, and L‐tryptophan	Control
2	Sodium chloride	NaCl
3	Humic acid	HA
4	L‐tryptophan	L‐TRP
5	Humic acid + sodium chloride	HA + NaCl
6	L‐tryptophan + sodium chloride	L‐TRP + NaCl
7	Humic acid + L‐tryptophan	HA + L‐TRP
8	Humic acid + L‐tryptophan + sodium chloride	HA + L‐TRP + NaCl

#### 2.2.2 Growth conditions of spinach plants and experimental design

A pot experiment was conducted to assess the effectiveness of HA and L‐TRP in mitigating the negative impacts of salt stress on spinach. The spinach was cultivated in a growth chamber set to 18 ± 1°C, with 50–65% relative humidity, and alternating 12 h of light and 12 h of darkness. The research was designed as a completely randomized trial with three repetitions for each treatment and three pots per repetition. Plastic pots measuring 19 × 17 × 14.5 cm were filled with 2 L of a peat and perlite mixture (3:1, v/v) as the growing medium. These pots were arranged on shelves within the growth chamber. Spinach seeds were sown at a density of 6–8 seeds per pot on February 24, 2023, and then irrigated immediately. Thinning was performed 15 days postsowing, leaving two plants per pot.

Salt, HA, and L‐TRP applications were started when 5–6 leaves were formed on the plants. Treatments were carried out twice a week for 6 weeks. Salt was applied to the soil; each pot was given 65 mL of NaCl solution via irrigation during every application. HA and L‐TRP were applied by foliar spray, ensuring both the upper and lower leaf surfaces were thoroughly coated with the solutions, covering all the leaves. For each application, 75 mL of HA and L‐TRP were administered to every pot. The control group's plant leaves were moistened with water. Throughout the experiment, the water status of the growing medium was monitored, and all plants were irrigated with equal volumes of water when needed. At the end of the experiment, the plants were harvested by removing them from the pots with their roots on May 10, 2023 (75 days after sowing). After harvest, plant growth parameters were determined, and chemical analyses were performed.

#### 2.2.3 Determination of plant growth parameters

Morphological measurements were made on six randomly chosen plants from each treatment group. Plant height (PH), leaf length (LL), root length (RL), leaf blade length (LBL), and leaf blade width (LBW) measurements were conducted using a ruler. The plant fresh weight (PFW) was determined with a precision balance. Following a 48‐h drying period in an oven at 65°C, the plant dry weight (PDW) was gauged using a precision scale. The number of leaves (NL) per plant was counted to determine the leaf count.

#### 2.2.4 Determination of mineral and biochemical contents

To determine the mineral content in spinach leaves, which includes phosphorus (P), potassium (K), calcium (Ca), magnesium (Mg), sodium (Na), sulfur (S), zinc (Zn), iron (Fe), nickel (Ni), chlorine (Cl), manganese (Mn), and copper (Cu), samples from each treatment were oven‐dried at 65°C for 48 h. These dried samples were then ground using a grinder (MC23200, Siemens, Germany). After grinding, the samples were made ready for analysis by microwave digestion method. The elemental analysis was conducted with an X‐RAY fluorescence spectrometer (XRF, Spectro XEPOS, Germany) at Kastamonu University's Central Research Laboratory.

The levels of chlorophyll, carotenoid, and xanthophyll in leaves were determined using modified methods originally described by Kukrić et al. (2012) and Chang et al. (2013). These contents were spectrophotometrically analyzed through absorption measurements ranging from 350 to 700 nm at 1.0 nm intervals and determined using specific formulas.

$$\text{Chlorophyll}\;a\;\left(\mathrm{mg}\;g^{-1}\right)=\left(13.7\times 665\right)-\left(5.76\times A649\right)/\text{mass}\times 200$$

$$\text{Chlorophyll}\;b\;\left(\mathrm{mg}\;g^{-1}\right)=\left(25.8\times A649\right)-\left(7.6\times A665\right)/\text{mass}\times 200$$

$$\text{Carotenoid}\;\left(\mathrm{mg}\;g^{-1}\right)=\left(4.7\times A440\right)-\left(0.263\times \text{CHLA}+\text{CHLB}\right)/\text{mass}\times 200$$

$$\text{Xanthophyll}\;\left(\text{lutein}\right)\;\left(\mathrm{mg}\;g^{-1}\right)=\left(11.51\times A480\right)-\left(20.61\times A495\right)/\text{mass}\times 200$$

The total polyphenol in spinach samples was measured following the Folin and Denis (1915) method. The absorbance of the resulting blue color was measured at 660 nm using a CE‐5502 Scanning Double Beam UV spectrophotometer after a 20‐min interval. The total polyphenol content was then determined from a standard curve of tannic acid at 0.1 mg mL^−1^.

The anthocyanin content in spinach samples was performed with the procedure defined by Sims and Gamon (2002). The content of anthocyanin in the samples was quantified with the following formula.

$$\text{Anthocyanin}\;\left(\text{μmol}\;{\mathrm{mL}}^{-1}\right)=0.08173\times A537-0.00697\times A647-0.002228\times A663$$

A× represents the absorbance value of the extract solution measured in a cuvette with a 1 cm path length at a specific wavelength denoted by ×.

The activity of polyphenol oxidase (PPO) in spinach samples was evaluated spectrophotometrically by observing the rise in absorbance at 420 nm with 4‐methyl catechol, following the methodology outlined by Kumar et al. (2008).

The free proline in leaf tissue was performed using the method recommended by Nazarli and Faraji (2011). The processed samples were thoroughly homogenized with 4 mL of toluene. Subsequently, the toluene layer was isolated, and its absorbance was recorded at 520 nm with a UV–visible spectrophotometer (Unico, S2100, USA). The concentration of proline was measured based on a standard curve.

The glycine betaine (GB) level was performed with the method suggested by Grieve and Grattan (1983). The samples included fully matured upper leaves of the plants. For the leaf extract preparation, 0.5 g of leaves were finely chopped in a 5 mL toluene–water solution (0.05% toluene) in 20 mL test tubes. The GB level was quantified by employing a standard curve created with varying concentrations of GB.

The absorbance of the prepared samples was determined at 532 and 600 nm to detect the malondialdehyde (MDA) content with the appropriate formula.

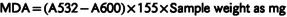




The level of hydrogen peroxide (H_2_O_2_) in spinach leaves was determined spectrophotometrically after reacting with potassium iodide, as stated by Alexieva et al. (2001). The reaction was carried out for 1 h in the dark, and the absorbance was recorded at 390 nm. The H_2_O_2_ level was determined with a standard curve of known H_2_O_2_ concentrations.

The relative water content (RWC) of spinach leaves was assessed using the García‐Mata and Lamattina (2001) method. The RWC was determined with the following equation.

$$\mathrm{RWC}\;\left(\%\right)=\left[\left(\mathrm{FW}-\mathrm{DW}\right)/\left(\mathrm{TW}-\mathrm{DW}\right)\right]\times 100$$

where FW, fresh weight, DW, dry weight, and TW, turgid weight.

The activities of antioxidant enzymes such as ascorbate peroxidase (APX), catalase (CAT), peroxidase (POD), and superoxide dismutase (SOD) were performed following the method described by Zhang et al. (2006). The activity of SOD was determined by its ability to inhibit the photochemical reduction of nitroblue tetrazolium (NBT). The reaction mixture, with a total volume of 3 mL, included 0.1 mL of enzyme extract, 50 mM phosphate buffer at pH 7.0, 2 μM riboflavin, 13 mM methionine, 75 μM NBT, and 10 μM EDTA. Test tubes containing the mixture were exposed to white fluorescent light for 15 minutes, after which the absorbance was measured at 560 nm.

All mineral and biochemical analyses were performed in triplicate for each treatment.

#### 2.2.5 Statistical analyses

The effects of independent variables on dependent variables (growth parameters and biochemical components) were examined by analysis of variance (ANOVA) using a one‐way design model including eight different treatments. The relationships between the obtained results were evaluated with Tukey's honestly significant difference (HSD) tests (*p* < .05). ANOVA analysis of the examined features was performed using JMP 16.0 software (SAS Institute Inc., Cary, North Carolina, USA). Heatmaps were used to compare parameters and present the results more effectively. Heatmaps were created through an online mapping platform (http://www.bioinformatics.com.cn/).

## 3 RESULTS AND DISCUSSION

### 3.1 Changes in growth parameters

Effects of treatments on growth parameters of spinach are shown in Table 2. The variance analysis revealed significant differences (*p* < .01) in terms of the parameters outlined in Table 2 for the various treatments. In the present study, plant growth parameters differed considerably according to salt, HA, and L‐TRP treatments. PH, PFW, and PDW varied with the treatments, ranging from 14.87 to 23.79 cm, 5.20 to 10.89 g, and 0.72 to 1.60 g, respectively. The greatest PH, PFW, and PDW were recorded in the HA + L‐TRP treatment. Regarding root length, the longest value was found in the L‐TRP treatment, with HA + L‐TRP and HA + L‐TRP + NaCl following. However, the lowest values with regards to the mentioned properties were detected in NaCl treatment. Among the treatments, maximum NL per plant was detected in HA + L‐TRP treatment with 11.75, and it was closely followed by L‐TRP, HA, HA + L‐TRP + NaCl, control, and L‐TRP + NaCl treatments. Maximum values for LL, LBL, and LBW were observed in the HA + L‐TRP treatment, while the minimum values for these leaf properties were noted in plants treated with NaCl. As anticipated, salt stress significantly reduced PH, PFW, PDW, NL, and overall leaf size in spinach. Conversely, the combined application of HA and L‐TRP under salt stress markedly mitigated the negative impacts of salt stress and improved plant growth. In comparison to the NaCl treatment, the HA + L‐TRP + NaCl treatment resulted in increases of 51.78% in PH, 77.50% in PFW, and 23.03% in NL. Furthermore, it was found that both individual and combined applications of HA and L‐TRP under nonsaline conditions positively influenced all measured growth parameters of spinach plants relative to the control.

**TABLE 2:** Effects of salt, humic acid, and L‐tryptophan treatments on growth parameters in spinach, where ±: standard deviation.

Treatment	Plant height (cm)	Root length (cm)	Plant fresh weight (g)	Plant dry weight (g)	Number of leaves (number plant^−1^)	Leaf length (cm)	Leaf blade length (cm)	Leaf blade width (cm)
Control	15.97 ± 1.34cd**	10.62 ± 0.56de**	7.06 ± 0.64d**	0.83 ± 0.09ef**	11.00 ± 1.19a**	11.01 ± 0.51de**	6.76 ± 0.56cd**	4.08 ± 0.65abc**
NaCl	14.87 ± 0.57d	9.49 ± 0.81e	5.20 ± 0.69e	0.72 ± 0.09f	9.25 ± 1.03b	10.44 ± 0.70e	6.61 ± 0.44d	3.68 ± 0.50c
HA	17.32 ± 0.92b	12.28 ± 0.75bc	8.80 ± 0.71bc	1.16 ± 0.10d	11.38 ± 0.92a	12.30 ± 0.95bc	7.51 ± 0.56bcd	4.44 ± 0.48abc
L‐TRP	17.06 ± 0.36bc	13.71 ± 0.77a	9.10 ± 0.81b	1.31 ± 0.09c	11.63 ± 0.92a	12.80 ± 0.62b	7.71 ± 0.60bc	4.81 ± 0.53ab
HA + NaCl	16.84 ± 0.67bc	11.36 ± 0.90 cd	7.91 ± 0.75 cd	0.88 ± 0.06e	10.50 ± 0.93ab	12.00 ± 0.63bcd	7.31 ± 0.64 cd	3.97 ± 0.57bc
L‐TRP + NaCl	17.86 ± 0.65b	12.16 ± 0.78bc	7.10 ± 0.72d	0.94 ± 0.05e	11.00 ± 0.93a	11.27 ± 0.71cde	8.46 ± 0.62ab	4.43 ± 0.64abc
HA + L‐TRP	23.79 ± 0.72a	12.95 ± 0.50ab	10.89 ± 0.74a	1.60 ± 0.06a	11.75 ± 1.03a	14.14 ± 0.64a	8.93 ± 0.90a	4.91 ± 0.58a
HA + L‐TRP + NaCl	22.57 ± 0.72a	12.70 ± 0.84ab	9.23 ± 0.67b	1.48 ± 0.06b	11.38 ± 0.92a	12.02 ± 0.71bcd	8.81 ± 0.58a	4.83 ± 0.65ab

*Note*: Different lowercase letters indicate differences between applications in terms of the properties examined.

Abbreviations: HA, humic acid; L‐TRP, L‐tryptophan.

** Significant at *p* < .01 level.

Like our findings, Kim et al. (2021) stated that salinity significantly reduced the growth of spinach plants. The adverse effect of salt stress on plant growth is well documented across various vegetables (Akladious & Mohamed, 2018; Alfosea‐Simón et al., 2020; Gabr et al., 2022; Hussein et al., 2014; Jamil et al., 2018; Kalyoncu et al., 2017; Meganid et al., 2015). Typically, heightened salinity is recognized for its detrimental impacts on plant growth because of ion toxicity, osmotic stress, nutritional imbalance, and oxidative stress (Munns & Tester, 2008). Salt stress may adversely influence the biochemical pathways involved in plant growth, limit water uptake, lower the net rate of photosynthesis, and significantly deplete the minerals available in the roots (Parida & Das, 2005). Delgado and Sánchez‐Raya (2007) attribute the reduction in root and shoot growth to the suppression of cytokinesis, cell expansion, and cell proliferation due to salt stress. Studies by Hafez et al. (2015), Naseri et al. (2021), and Turan et al. (2022) have reported that HA treatment significantly boosts plant growth properties like PFW, PDW, and NL in spinach, compared to untreated controls, aligning with our observations. Humic substances are recognized to enhance plant growth by influencing mineral nutrient uptake, stimulating photosynthesis, increasing microbial populations, regulating enzyme activities, and functioning as plant hormones, as noted by Gholami et al. (2013). Furthermore, Aydin et al. (2012), Meganid et al. (2015), Kaya et al. (2018), and Gabr et al. (2022) have stated that HA treatments promote plant growth in both saline and nonsaline environments. Previous research has documented significant growth improvements in various vegetables under salt stress following HA application (Akladious & Mohamed, 2018; Çimrin et al., 2010; Kalyoncu et al., 2017; Khaled & Fawy, 2011; Osman & Rady, 2012). HA is known to mitigate stress effects under abiotic stress conditions, enhancing plant stress resistance (Hanafy et al., 2010), and its application is beneficial for salinity tolerance by encouraging nutrient absorption and bolstering the antioxidant system (Aydin et al., 2012). Studies have demonstrated that the application of HA can improve a plant's resilience to salt stress by boosting nutrient uptake and minimizing the absorption of toxic elements (Liu & Cooper, 2002). The use of L‐TRP has been shown to enhance the growth of various vegetables, including mung bean (Zahir et al., 2010), onion (Hussein et al., 2014), okra (Mustafa et al., 2016), and red pepper (Jamil et al., 2018). Furthermore, exogenous L‐TRP treatments depending on salinity positively affected the growth of different vegetables (Alfosea‐Simón et al., 2020; Hussein et al., 2014; Jamil et al., 2018; Zahir et al., 2010). The growth‐promoting effects of L‐TRP could be attributed to its direct absorption by plant roots and the subsequent transformation into auxin metabolites, which may assist the plant in coping with salinity.

### 3.2 Changes in mineral contents

The current study found that the impacts of salt, HA, and L‐TRP applications on the mineral content of spinach, with the exception of copper, were significant (*p* < .01). The mineral levels considerably changed depending on the treatments. When different treatments were examined, it was found that plants treated with NaCl showed the maximum contents of Na, K, Cl, and Ni. Maximum values in terms of Mg and Mn were found in L‐TRP + NaCl treatment. HA + NaCl treatment had the highest P, Ca, S, and Zn contents. With respect to Fe content, control treatment took the first place with 2830 mg kg^−1^. Salinity reduced the absorption of Fe compared to the control. The contents of K, Mg, P, Ca, Cl, S, Zn, and Ni were found to be the lowest in the L‐TRP application. In the control application, plants exhibited the lowest Na concentration. The lowest Fe content was detected in HA + NaCl, L‐TRP + NaCl, NaCl, and HA + L‐TRP + NaCl treatments. The lowest Mn content was determined in HA, L‐TRP, and HA + L‐TRP treatments. In the current research, it was found that NaCl treatment raised the element contents of spinach, except Fe, compared to the control. As might be expected, Na and Cl accumulated strongly in spinach plants after NaCl application. In comparison to the control, Na level was 3.9 times higher and Cl content was 3.7 times higher in the NaCl treatment. Salinity decreased plant growth due to mineral imbalance. HA and L‐TRP suppressed the Na and Cl increase caused by salt stress. HA treatment under salt stress (HA + NaCl) significantly enhanced essential mineral contents such as Mg, P, and Ca compared to the NaCl treatment. Likewise, L‐TRP treatment under salt stress (L‐TRP + NaCl) considerably increased concentrations of Mg and Mn compared to salt application alone (Table 3).

**TABLE 3:** Effects of salt, humic acid, and L‐Tryptophan treatments on mineral contents in spinach, where ±: standard deviation.

Treatment	K	Mg	P	Ca	Na	Cl	S	Fe	Mn	Zn	Ni	Cu
	mg kg^−1^, DW											
Control	92,460 ± 3544d**	12,861 ± 1032cd**	5834 ± 113bc**	17,231 ± 211d**	4840 ± 201f**	5442 ± 211d**	6726 ± 317bc**	2830 ± 218a**	104.31 ± 10.2cd**	120.60 ± 10.1b**	33.54 ± 2.1abc**	13.81 ± 1.1^ns^
NaCl	130,721 ± 5699a	20,661 ± 1088b	6625 ± 208b	24,680 ± 1145b	18,781 ± 2012a	20,353 ± 2398a	9878 ± 1135a	651 ± 50d	135.85 ± 5.4ab	143.80 ± 10.4a	37.90 ± 3.1a	14.61 ± 2.0
HA	74,260 ± 4124e	14,346 ± 325c	5048 ± 1077 cd	14,562 ± 469de	8866 ± 209e	3848 ± 236de	5879 ± 196 cd	1551 ± 200c	84.71 ± 4.2d	83.73 ± 10.6 cd	27.49 ± 0.9 cd	11.91 ± 0.5
L‐TRP	62,462 ± 2551f	10,821 ± 119d	4237 ± 239d	11,543 ± 1023f	13,680 ± 1047 cd	2729 ± 218e	5026 ± 554d	1788 ± 100bc	86.71 ± 4.3d	72.91 ± 2.9d	26.82 ± 4.1d	12.30 ± 0.6
HA + NaCl	114,523 ± 2688b	25,483 ± 426a	7878 ± 366a	30,461 ± 1421a	16,545 ± 1019ab	14,265 ± 1241bc	10,270 ± 1324a	603 ± 51d	126.57 ± 20.4bc	152.92 ± 5.9a	32.59 ± 1.5a‐d	14.27 ± 1.1
L‐TRP + NaCl	102,406 ± 2141c	27,246 ± 1029a	5270 ± 141 cd	21,470 ± 1133c	14,775 ± 359bc	12,682 ± 1077c	6692 ± 269bc	643 ± 22d	156.8 ± 10.9a	103.81 ± 3.7bc	28.54 ± 2.2bcd	14.89 ± 1.3
HA + L‐TRP	68,359 ± 1698ef	11,480 ± 409d	4460 ± 209d	13,675 ± 398ef	11,547 ± 1032de	3047 ± 211de	5372 ± 297cd	1953 ± 103b	88.90 ± 4.1d	86.53 ± 4.8 cd	28.68 ± 2.1bcd	12.64 ± 2.0
HA + L‐TRP + NaCl	115,489 ± 5589b	22,428 ± 1171b	5739 ± 312bc	26,729 ± 2141b	14,263 ± 209bc	16,347 ± 399b	7542 ± 289b	825 ± 55d	128.52 ± 10.6bc	114.56 ± 10.8b	34.30 ± 1.3ab	13.76 ± 1.4

*Note*: ns: nonsignificant (*p* > .05). Different lowercase letters indicate differences between applications in terms of the properties examined.

Abbreviations: Ca, calcium; Cl, chlorine; Cu, copper; DW, dry weight; Fe, iron; HA, humic acid; K, potassium; L‐TRP, L‐tryptophan; Mg, magnesium; Mn, manganese; Na, sodium; Ni, nickel; P, phosphorus; S, sulfur; Zn, zinc.

** Significant at *p* < .01 level.

Salinity is known to reduce the net photosynthetic rate and nutrient uptake (Parida & Das, 2005). The study conducted by Kim et al. (2021) indicated that salinity adversely impacts the nutritional quality of spinach, notably increasing the Na content while decreasing K, Ca, and Fe levels. Likewise, Di Martino et al. (2003) detected an important rise in Na level in spinach under saline conditions. Sheikhi and Ronaghi (2012) found that salinity led to lower Fe, K, and Mg levels in spinach, but higher Na, P, N, and CI levels. As salinity rises, the absorption of Na and Cl from the soil increases, which may hinder the uptake of other vital nutrients like N, K, P, and Ca (Safdar et al., 2019; Shrivastava & Kumar, 2015). Generally, an excess of Na in the soil can competitively inhibit the uptake of other cations like Ca, K, Mg, and Fe (Assaha et al., 2017). The adverse impact of salt on the uptake of mineral elements may be due to the soil solution's low osmotic potential, which results from heightened NaCl concentration. A key indicator of salt tolerance is the K^+^/Na^+^ ratio (Munns & Tester, 2008). Maintaining a high K^+^/Na^+^ ratio in plants depending on saline conditions is vital for improving the salt tolerance index (Ouni et al., 2014). Increased salinity in the root zone lowers the K^+^/Na^+^ ratio, leading to an ion imbalance. This imbalance in the K^+^/Na^+^ ratio impairs plant growth and physiological functions, including photosynthesis.

In this study, K and Ca contents were found to be higher than control plants under salt stress conditions (NaCl treatment only). Under salt stress, plants increase K uptake to counteract the osmotic pressure caused by excessive Na ions. To mitigate the toxic effects of high Na levels, plants often enhance K uptake to maintain a favorable Na/K ratio. This helps in stabilizing enzyme activities and maintaining cellular functions. Under salt stress, increased Ca uptake helps in maintaining membrane integrity and preventing ion leakage. Increased Ca levels help in mitigating the detrimental effects of Na accumulation.

Hafez et al. (2015) stated that HA treatment raised the levels of N, P, and K in spinach compared to the control. In another study, significant increases were obtained in the Cu, Zn, and Mn values of spinach with the application of HA (Gülser & Ayaș, 2016). Similarly, Turan et al. (2022) found that foliar treatments of humic substances markedly improved the N, P, K, Mg Ca, Cu, S, Zn, Mn, and B values of spinach compared to the control. Al‐Falahi et al. (2022) highlighted that HA treatment not only raised the Ca and Mg concentrations but also reduced Na and Cl levels in broccoli depending on salt stress. Additionally, Aydin et al. (2012) stated that HA treatment boosted the N and P contents of bean plants depending on salinity. Gabr et al. (2022) found that HA treatment depending on salt stress raised the levels of N, P, K, and Ca in lettuce while reducing Na concentration. Kalyoncu et al. (2017) found that HA application under salt stress negatively affected the uptake of Na, Mg, and Mn in mung bean, while it enhanced the uptake of K, Ca, and Zn. Khaled and Fawy (2011) demonstrated that foliar application of HA under salt stress raised the absorption of N, P, K, Ca, and Mg in corn. Turhan et al. (2020) noted that HA addition under nonsaline conditions raised the values of N, P, K, Ca, Mg, Fe, and Zn in onion, while decreasing Cl content. Moreover, HA application under salt stress boosted the contents of K, Ca, and N. Osman and Rady (2012) and El‐Sarkassy et al. (2017) observed that HA application under salt stress significantly lowered Na content in pea and pepper plants, while elevating N, P, and K levels compared to salt treatment alone. Çimrin et al. (2010) found that HA treatment importantly raised the contents of N, P, K, Mg, Ca, S, Mn, and Cu in pepper depending on moderate salinity. Conversely, HA addition negatively affected the uptake of Na, Zn, and Fe. Kaya et al. (2018) found that HA treatment decreased Na content of maize under saline conditions, whereas it increased values of N, Ca, P, and K. Akladious and Mohamed (2018) found that HA application under salt stress decreased Na content of pepper plants compared to salt application alone and it enhanced N, P, and K contents. HA effectively increases plant nutrient uptake, especially in saline soil. HA can stabilize soil pH in the root zone, thereby aiding nutrient absorption. HA increases microbial activity and nutrient availability in the soil. It also serves as a natural chelator, which increases Fe availability. The increase in mineral uptake following HA application may be due to increased plasma membrane permeability, soil water retention, microbial activity, and the solubility of Zn, Fe, Mg, and Cu in the soil, as noted by David et al. (1994). HA could significantly reduce Na levels due to K supplementation. Alfosea‐Simón et al. (2020) stated that L‐TRP treatment under salt stress markedly raised Na and Cl contents in tomato plants, while it substantially lowered K levels compared to salt treatment alone. The variations observed in studies can be due to factors like plant species, cultivar, growth stage of the plant, application dose, application method, growing media, environmental conditions, and laboratory methods used.

### 3.3 Changes in photosynthetic pigments

The study revealed statistically significant variations (*p* < .01) among treatments concerning chlorophyll *a*, chlorophyll *b*, total chlorophyll, carotenoid, and lutein values. The content of chlorophyll *a*, chlorophyll *b*, and total chlorophyll was markedly affected by the various treatments. Total chlorophyll content in this study varied from 2.08 to 2.69 mg g^−1^. The HA + L‐TRP treatment yielded the maximum values of chlorophyll *a*, chlorophyll *b*, and total chlorophyll, while the NaCl treatment resulted in the lowest values. Spinach treated with NaCl consistently showed a reduction in chlorophyll content. HA + L‐TRP treatment had the maximum values regarding carotenoid and lutein values. However, the contents of carotenoid and lutein were found to be lowest in the NaCl treatment. Salt stress significantly reduced the values of chlorophyll *a*, chlorophyll *b*, total chlorophyll, carotenoid, and lutein in spinach. Conversely, the combined application of HA and L‐TRP, in response to salt stress, alleviated the adverse impacts of salinity on these characteristics. In comparison to the NaCl treatment, the HA + L‐TRP + NaCl treatment resulted in a 19.71% and 17.74% increase in total chlorophyll and carotenoid, respectively. Furthermore, the study revealed that individual and combined applications of HA and L‐TRP under nonsaline conditions had a positive effect on total chlorophyll, carotenoid, and lutein contents of spinach plants compared to the control (Table 4).

**TABLE 4:** Effects of salt, humic acid, and L‐tryptophan treatments on photosynthetic pigments in spinach, where ±: standard deviation.

Treatment	Chlorophyll *a*	Chlorophyll *b*	Total chlorophyll	Carotenoid	Lutein
	mg g^−1^, FW				
Control	0.87 ± 0.02b**	1.47 ± 0.02e**	2.35 ± 0.02e**	11.72 ± 0.81cd**	0.74 ± 0.08 cd**
NaCl	0.80 ± 0.02c	1.28 ± 0.01f	2.08 ± 0.01f	11.33 ± 0.88d	0.59 ± 0.08d
HA	0.95 ± 0.06a	1.61 ± 0.02b	2.56 ± 0.03b	14.81 ± 0.84a	0.93 ± 0.09bc
L‐TRP	0.95 ± 0.01a	1.54 ± 0.01d	2.49 ± 0.01c	14.17 ± 0.83ab	1.03 ± 0.10ab
HA + NaCl	0.81 ± 0.01c	1.58 ± 0.01c	2.39 ± 0.01d	12.30 ± 0.76bcd	0.76 ± 0.07 cd
L‐TRP + NaCl	0.87 ± 0.01b	1.45 ± 0.01e	2.32 ± 0.01e	12.06 ± 0.80 cd	0.79 ± 0.09c
HA + L‐TRP	0.96 ± 0.02a	1.72 ± 0.02a	2.69 ± 0.01a	15.25 ± 0.84a	1.19 ± 0.08a
HA + L‐TRP + NaCl	0.88 ± 0.02b	1.61 ± 0.01b	2.49 ± 0.01c	13.34 ± 0.80abc	0.85 ± 0.08bc

*Note*: Different lowercase letters indicate differences between applications in terms of the properties examined.

Abbreviations: FW, fresh weight; HA, humic acid; L‐TRP, L‐tryptophan.

** Significant at *p* < .01 level.

Many researchers stated that the chlorophyll concentration in spinach (chlorophyll *a*, chlorophyll *b*, and total chlorophyll content) is importantly decreased by high salinity compared to control groups (Di Martino et al., 2003; Ratnakar & Rai, 2013; Seven & Sağlam, 2020), aligning with the current study's findings. Akladious and Mohamed (2018) found that salt stress led to a notable reduce in chlorophyll *a* and *b*, and carotenoid levels in pepper relative to unstressed controls. Kim et al. (2021) highlighted that salinity lowered the carotenoid and lutein levels in spinach, corroborating our results. Similarly, salinity has been shown to significantly diminish chlorophyll content in bean (Aydin et al., 2012; Meganid et al., 2015), corn (Kaya et al., 2018), tomato (Alfosea‐Simón et al., 2020), and lettuce (Gabr et al., 2022). Degradation of chlorophyll is regarded as a sign of oxidative damage (Ahmad et al., 2018), and oxidative stress from salinity can harm the cell membrane, leading to chlorophyll breakdown (Apel & Hirt, 2004). The reduction in chlorophyll content associated with salinity is attributed to the negative impacts of accumulated ions like Na and Cl on the synthesis of photosynthetic pigments. The decrease in photosynthetic pigments in plants under salt stress may be due to the decrease in the intake of minerals such as Mg and Fe, which are necessary for chlorophyll biosynthesis, the increase in the activity of chlorophyllase, which is a chlorophyll‐degrading enzyme, and decrease in chlorophyll synthesis (Jaleel et al., 2008). The reduction in chlorophyll *a*, chlorophyll *b*, and carotenoid levels may result from the degradation of chloroplasts and photosynthetic pigments, as suggested by Moradi and Ismail (2007). Additionally, an increase in proline synthesis might contribute to lower chlorophyll under stress conditions. The drop in chlorophyll concentration under salt stress leads to suppressed photosynthetic activity (Amuthavalli & Sivasankaramoorthy, 2012).

On the other hand, HA treatment was shown to significantly boost chlorophyll *a* and *b*, total chlorophyll, and total carotenoid values in spinach compared to the control group, aligning with our findings (Hafez et al., 2015; Naseri et al., 2021; Turan et al., 2022). Additionally, HA application under salinity led to higher chlorophyll *a*, chlorophyll *b*, and carotenoid levels in pea and pepper than with salt treatment alone (Akladious & Mohamed, 2018; Osman & Rady, 2012). The exogenous treatment of HA also markedly increased chlorophyll levels in lettuce, common bean, and maize under both standard and saline conditions (Gabr et al., 2022; Kaya et al., 2018; Meganid et al., 2015). Additionally, HA treatment elevated carotenoid levels in pepper (Aminifard et al., 2012). In our research, applying HA in both normal and saline environments resulted in increased chlorophyll levels in spinach. HA has been shown to stimulate chlorophyll synthesis by promoting nitrogen uptake, as reported by Xu et al. (2012). The boost in photosynthetic pigments due to HA can be linked to a drop in soil pH and a rise in soil organism activity (Akladious & Mohamed, 2018). Hussein et al. (2014) found a rise in carotenoid content of onion due to salinity and L‐TRP treatment enhanced chlorophyll and carotenoid contents compared to the control. Jamil et al. (2018) reported that L‐TRP raised chlorophyll *a*, chlorophyll *b*, and carotenoid levels in red pepper under saline and nonsaline conditions, corroborating our results. However, Gerekli (2015) indicated that L‐TRP application under salinity stress did not markedly alter chlorophyll *a*, chlorophyll *b*, and total chlorophyll contents of pepper plants. L‐TRP is a precursor to the plant hormone indole‐3‐acetic acid (IAA), an auxin. Auxins can promote the synthesis of chlorophyll, leading to increased chlorophyll content in leaves. This enhances the plant's photosynthetic capacity. L‐TRP can help plants cope with stress conditions by enhancing the production of photosynthetic pigments. This is often due to its role in synthesizing secondary metabolites. In addition to chlorophyll, L‐TRP can influence the levels of carotenoids, which are important for protecting the photosynthetic apparatus from oxidative damage.

### 3.4 Changes in antioxidant compounds and oxidative stress

Table 5 shows significant differences in the contents of polyphenol, anthocyanin, PPO, proline, GB, MDA, RWC (*p* < .01), and H_2_O_2_ (*p* < .05) among the treatments with salt, HA, and L‐TRP. The levels of polyphenol, anthocyanin, and PPO in the HA + L‐TRP treatment were significantly higher compared to other treatments. Conversely, these properties were observed to be the lowest in the NaCl treatment. Depending on salt, HA, and L‐TRP treatments investigated in the study, proline content varied from 28.61 (control) to 39.04 μmol g^−1^ (HA + L‐TRP + NaCl). Among the treatments, maximum GB contents were detected in HA, L‐TRP, and HA + L‐TRP treatments, while minimum GB contents were recorded in NaCl, HA + NaCl, and L‐TRP + NaCl treatments. MDA content ranged from 137.75 (HA + L‐TRP) to 185.79 μmol g^−1^ (NaCl). H_2_O_2_ content was the highest in NaCl treatment, whereas it was the lowest in L‐TRP and L‐TRP + NaCl treatments. The highest RWC values were observed in treatments with HA, L‐TRP, HA + NaCl, L‐TRP + NaCl, HA + L‐TRP, and HA + L‐TRP + NaCl, which were statistically grouped together. Conversely, the NaCl treatment exhibited the lowest RWC. In the present study, significant increases were observed in proline, MDA, and H_2_O_2_ levels of spinach with salinity. Compared with the control, NaCl treatment enhanced proline, MDA, and H_2_O_2_ contents by 27.54%, 10.12%, and 25.77%, respectively. Conversely, spinach experienced a significant decrease in polyphenol, anthocyanin, PPO, GB, and RWC levels due to salinity. Furthermore, it was found that both separate and combined treatments with HA and L‐TRP in the absence of salinity positively influenced the polyphenol, anthocyanin, PPO, proline, GB, and RWC levels in spinach plants relative to the control. Under saline conditions, the combined use of HA and L‐TRP markedly enhanced these properties. Compared to the NaCl treatment, HA + L‐TRP + NaCl treatment increased polyphenol content and RWC of spinach by 54.90% and 38.49%, respectively.

**TABLE 5:** Effects of salt, humic acid, and L‐tryptophan treatments on antioxidant compounds and oxidative stress in spinach, where ±: standard deviation.

Treatment	Total polyphenol (g 100 g^−1^, FW)	Anthocyanin (μmol mL^−1^, FW)	PPO (U mg protein^−1^, FW)	Proline (μmol g^−1^, FW)	GB (μg g^−1^, FW)	MDA (μmol g^−1^, FW)	H_2_O_2_ (μmol g^−1^, FW)	RWC (%, DW)
Control	46.91 ± 2.55c**	2.75 ± 0.09c**	2.15 ± 0.07 cd**	28.61 ± 0.81c**	4.66b ± 0.08**	168.72 ± 8.03ab**	124.66 ± 16.4ab*	80.47 ± 8.25ab**
NaCl	40.64 ± 4.21c	2.53 ± 0.08d	1.73 ± 0.08e	36.49 ± 0.91a	3.88 ± 0.08d	185.79 ± 7.90a	156.79 ± 18.2a	66.75 ± 3.29b
HA	61.08 ± 4.06ab	2.87 ± 0.08bc	2.35 ± 0.08bc	32.16 ± 0.75b	5.45 ± 0.09a	142.36 ± 18.2b	133.27 ± 21.6ab	87.15 ± 3.50a
L‐TRP	66.88 ± 3.14a	2.96 ± 0.12ab	2.66 ± 009a	33.34 ± 0.87b	5.26 ± 0.09a	146.49 ± 8.42b	116.69 ± 14.3b	90.71 ± 8.12a
HA + NaCl	57.27 ± 3.21b	2.84 ± 0.08bc	2.28 ± 0.08bcd	36.96 ± 1.62a	3.98 ± 0.08d	160.46 ± 18.4ab	125.43 ± 16.4ab	83.28 ± 7.94a
L‐TRP + NaCl	60.96 ± 4.00ab	2.82 ± 0.08bc	2.42 ± 0.08b	36.59 ± 1.63a	3.90 ± 0.07d	152.75 ± 16.4ab	115.61 ± 16.5b	85.51 ± 4.27a
HA + L‐TRP	69.19 ± 3.62a	3.13 ± 0.08a	2.76 ± 0.08a	38.31 ± 1.68a	5.33 ± 0.13a	137.75 ± 16.5b	120.41 ± 16.2ab	92.44 ± 8.24a
HA + L‐TRP + NaCl	62.95 ± 4.75ab	2.86 ± 0.08bc	2.10 ± 0.14d	39.04 ± 1.61a	4.43 ± 0.09c	155.30 ± 16.1ab	118.70 ± 16.1ab	87.23 ± 2.75a

*Note*: Different lowercase letters indicate differences between applications in terms of the properties examined.

Abbreviations: DW, dry weight; FW, fresh weight; GB, glycine betaine; HA, humic acid; H_2_O_2_, hydrogen peroxide; L‐TRP, L‐tryptophan; MDA, malondialdehyde; PPO, polyphenol oxidase; RWC, relative water content.

* Significant at *p* < .05 level.

** Significant at *p* < .01 level.

High salinity levels lead to significant increases in the production of ROS (Ventura et al., 2014). In order to prevent an overproduction of ROS, plants produce nonenzymatic small molecules, including flavonoids, phenolic acids, and proanthocyanidins, as reported by Waśkiewicz et al. (2013). As stated by Amarowicz and Weidner (2009), phenolic compounds are considered antioxidant molecules that are crucial for scavenging ROS. Research has shown that the antioxidant capacity, total phenolic content, and levels of polyphenols and phenolic acids in spinach decrease progressively with increased salinity (Tareq et al., 2021). Consistent with our results, Aslam et al. (2016) noted an elevation in the total phenolic content of spinach plants following the application of HA, in contrast to the control group. This augmentation may be due to HA's role in boosting antioxidants that neutralize ROS, associated with oxidative stress. Another study showed that applying HA increased the antioxidant activity, as well as the total phenolic and carotenoid levels in pepper (Aminifard et al., 2012). Akladious and Mohamed (2018) observed that salt stress decreased the anthocyanin content in pepper relative to the control. Nevertheless, the application of HA under salt stress conditions raised the anthocyanin content in comparison to both the control and the plants subjected to salt stress alone, confirming the findings of this study.

It is known that proline and GB protect plant tissues from osmotic stress (Zhao et al., 2009). In line with this, Ratnakar and Rai (2013) highlighted an elevation in proline levels in spinach when subjected to salt stress. Likewise, Di Martino et al. (2003) found that salinity markedly increased the levels of proline and GB in spinach when compared to the control. Kim et al. (2021) also noted a rise in GB levels in spinach correlating with higher NaCl doses. In the studies conducted by Aydin et al. (2012) and Kaya et al. (2018), salinity significantly increased proline content of bean and maize plants, while exogenous application of HA reduced proline content depending on salinity. Conversely, Osman and Rady (2012) emphasized that HA treatment under salt stress significantly enhanced free proline content of peas compared to salt application alone. Alfosea‐Simón et al. (2020) stated that salinity notably raised the proline levels in tomato plants when compared to the control, whereas the application of L‐TRP depending on salt stress significantly reduced the proline levels relative to the salinity treatment alone. The increase in proline levels in plants under salt stress is indicative of their salinity tolerance, as suggested by Munns and Tester (2008). Plants deal with this adversity by enhancing proline accumulation. The regulation of osmotic stress involves elevating the levels of osmolytes like proline (Ahmad et al., 2018). Proline is recognized for its role in osmoregulation, functioning as an osmoprotectant and a free radical scavenger (Yildiz & Terzi, 2013). Proline is advantageous under salt stress conditions as it reduces the water potential inside the cell and prevents intracellular loss (Mahajan & Tuteja, 2005). Proline serves as a stress marker and helps protect plants from damage caused by stress (Kaur & Sirhindi, 2015). The reduction in proline levels in plants treated with HA may be attributed to its role in counteracting the detrimental effects of salinity.

This study's findings align with those of Kaya et al. (2018), who observed that salt stress markedly elevated the H_2_O_2_ and MDA levels in maize plants, whereas the external application of HA notably decreased these levels under saline conditions. Contrary to our observations, L‐TRP elevated the MDA levels in onion plants in both saline and nonsaline environments (Hussein et al., 2014). In the study conducted by Gerekli (2015), L‐TRP applications under salinity significantly reduced MDA and H_2_O_2_ contents in pepper, aligning with the current study's findings. The rise in free radicals under salinity conditions results in the breakdown of membrane lipids and the production of MDA (Bernstein et al., 2010). The level of lipid peroxidation is indicated by the content of MDA, which serves as a marker for membrane damage due to ROS in stress conditions. Damage to cell membrane due to salt stress is associated with oxidative stress resulting from lipid peroxidation. HA mitigates lipid peroxidation and ROS levels by influencing the activity of certain enzymes within the antioxidant defense system (Lotfi et al., 2015). Indeed, our study found that HA curbed the rise in MDA under both saline and normal conditions. The accumulation of H_2_O_2_ is a response in plants to various biotic and abiotic factors.

In line with this study's findings, notable decreases in RWC were observed in pepper and tomato plants depending on salinity, as reported by Akladious and Mohamed (2018) and Alfosea‐Simón et al. (2020), respectively. The research by Akladious and Mohamed (2018) showed that the use of HA elevated the RWC in pepper under salt stress. Previous studies indicated that the external treatment of L‐TRP under salt stress resulted in an increase in RWC of pepper and tomato plants (Alfosea‐Simón et al., 2020; Jamil et al., 2018), which aligns with our observations. The reduction in RWC under salt stress is probably attributable to the build‐up of toxic ions within the plant's root zone. HA enhances the water retention capacity of the soil and maintains the water potential of the soil by absorbing more water by the root, thereby increasing the RWC of the leaves. Plants treated with HA are more adept at preserving leaf water content during osmotic stress (Canellas et al., 2015). In our study, HA and L‐TRP contributed to the increase in RWC, which could reduce the severity of salt stress on spinach plants.

### 3.5 Changes in antioxidant enzyme activities

The impact of salt, HA, and L‐TRP treatments on antioxidant enzyme activities of spinach was statistically significant (*p* < .01). APX, POD, and SOD activities in HA + L‐TRP treatment were significantly higher than other treatments. The maximum CAT activity was found in L‐TRP treatment. Contrarily, the activity of antioxidant enzymes was lowest in the NaCl treatment. Salt stress markedly decreased the antioxidant enzyme activities in spinach. Conversely, both individual and combined applications of HA and L‐TRP under salt stress elevated these enzyme activities. Additionally, it was observed that HA and L‐TRP treatments resulted in higher enzyme activity levels compared to the control, as shown in Table 6.

**TABLE 6:** Effects of salt, humic acid, and L‐tryptophan treatments on antioxidant enzyme activities (APX, CAT, POD, and SOD) in spinach, where ±: standard deviation.

Treatment	APX	CAT	POD	SOD
	EU mg protein^−1^, FW			
Control	5.51 ± 0.17d**	4.37 ± 0.16e**	6.41 ± 0.14c**	88.66 ± 8.10bcd**
NaCl	4.25 ± 0.14e	2.67 ± 0.17f	4.60 ± 0.21d	70.05 ± 8.10d
HA	6.17 ± 0.20c	5.21 ± 0.09c	7.33 ± 0.19b	96.97 ± 7.87abc
L‐TRP	6.74 ± 0.18b	6.40 ± 0.12a	8.07 ± 0.16a	107.96 ± 7.89ab
HA + NaCl	5.52 ± 0.07d	4.86 ± 0.17d	4.66 ± 0.12d	74.47 ± 8.10d
L‐TRP + NaCl	5.87 ± 0.14 cd	5.06 ± 0.08 cd	4.94 ± 0.14d	80.98 ± 7.80 cd
HA + L‐TRP	7.28 ± 0.14a	6.07 ± 0.11b	8.34 ± 0.20a	114.40 ± 8.32a
HA + L‐TRP + NaCl	5.92 ± 0.16c	4.88 ± 0.18d	4.77 ± 0.10d	86.59 ± 9.63 cd

*Note*: Different lowercase letters indicate differences between applications in terms of the properties examined.

Abbreviations: APX, ascorbate peroxidase; CAT, catalase; FW, fresh weight; HA, humic acid; L‐TRP, L‐tryptophan; POD, peroxidase; SOD, superoxide dismutase.

** Significant at *p* < .01 level.

Contrary to our findings, a raise in POD and SOD levels in spinach plants subjected to salt stress was reported (Seven & Sağlam, 2020). Similarly, Kaya et al. (2018) emphasized that salt stress markedly elevated the levels of CAT, POD, and SOD in maize. Nonetheless, the exogenous application of HA importantly diminished the activities of CAT, POD, and SOD in both saline and nonsaline environments. In another study, HA application increased APX activity in plants exposed to salinity (Kesba & El‐Beltagi, 2012). Stimulation of CAT activity through HA application was also stated (Cordeiro et al., 2011). HA may be effective in increasing antioxidant enzyme activities. In the research conducted by Gerekli (2015), L‐TRP applications under salt stress increased CAT and POD activities of pepper plants, which agreed with the results in the present research. According to our results, HA and L‐TRP under both normal and saline conditions were observed to have positive effects on antioxidant enzyme activities. This research suggests that HA and L‐TRP may induce antioxidant enzyme activities to scavenge H_2_O_2_. Excessive ROS production under salt stress results in oxidative stress, which detrimentally affects the cell membrane. Plants exposed to stress conditions respond at the molecular level by increasing enzymatic (such as catalase, ascorbate peroxidase, superoxide dismutase, peroxidase, and glutathione reductase) or nonenzymatic (such as phenolics, carotenoids, tocopherols, and ascorbic acid) antioxidant levels (Sevengor et al., 2011). These antioxidants, which take part in the elimination of free radicals such as H_2_O_2_ formed in the tissues because of exposure to stress and controlling the ROS level, are also known as stress enzymes, and it is thought that there is a positive relationship between the high level of their activity and plant's tolerance to stress. Scavenging ROS by increasing the activity of antioxidant enzymes can improve the salt tolerance of plants (Khalid & Aftab, 2020). The activities of enzymatic antioxidants such as CAT, POD, APX, and SOD are linked to the salinity tolerance of the plant because these antioxidants take part in neutralizing free radical derivatives generated in plant tissues because of oxidative stress caused by salt (Ashraf, 2009). SOD acts as the primary defense against ROS (Ahmad & Wani, 2013). Elevated POD activity lowers H_2_O_2_ levels in cells, enhancing membrane stability (Kaya et al., 2018). APX decreases the concentrations of H_2_O_2_, thus playing a crucial role in safeguarding the plant during salt stress (Anjum et al., 2011).

### 3.6 Heatmap analysis

**FIGURE 1:**
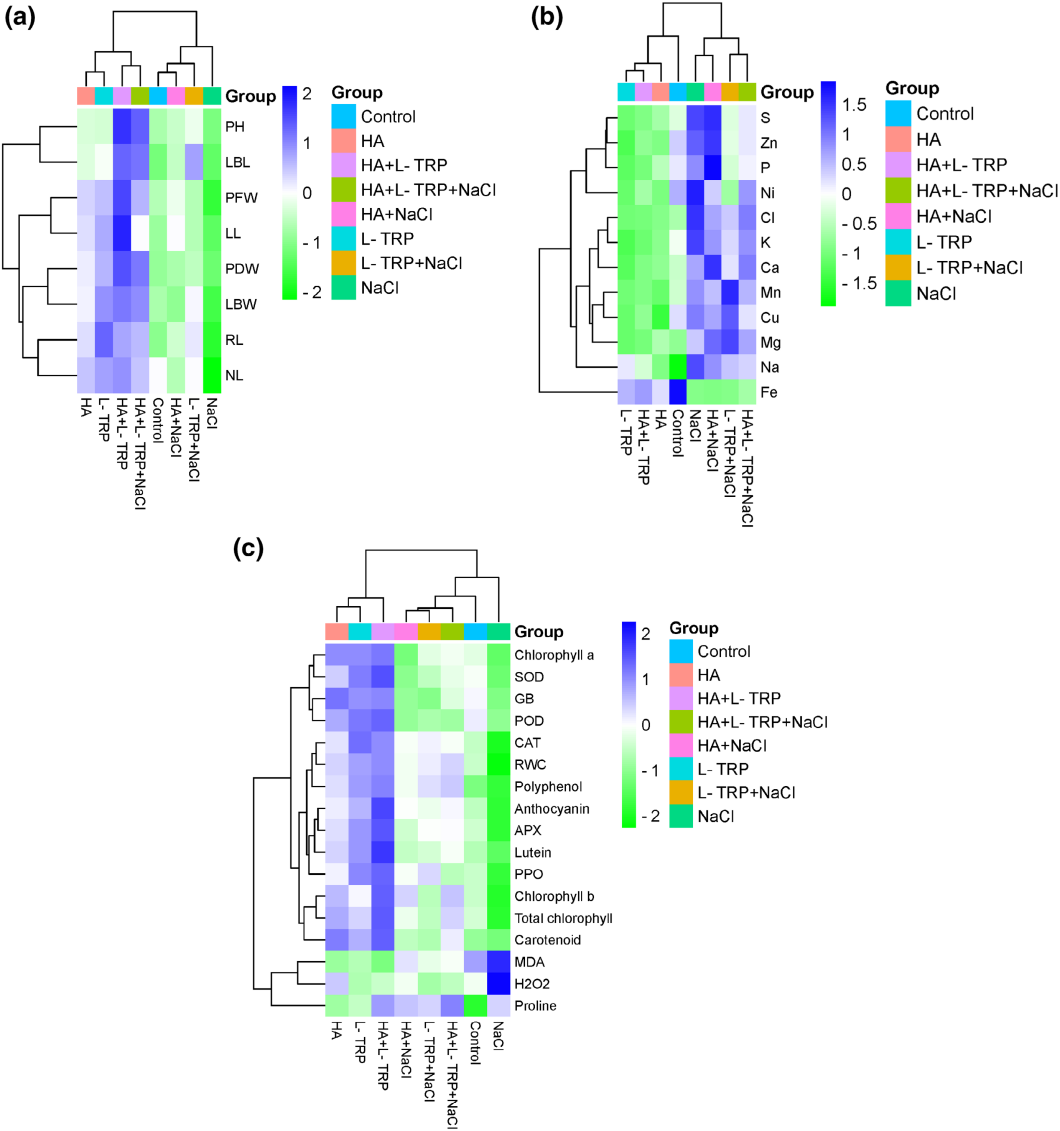
Heatmap showing various properties of spinach under NaCl stress conditions. The right side shows the features examined, and the bottom side shows the treatments. Blue color indicates positive correlation and green color indicates negative correlation. Dendrograms on the top and left show parameters grouped with similar features. APX, ascorbate peroxidase; Ca, calcium; CAT, catalase; Cl, chlorine; Cu, and copper; Fe, iron; GB, glycine betaine; H_2_O_2_, hydrogen peroxide; HA + L‐TRP + NaCl, humic acid + L‐tryptophan + sodium chloride; HA + L‐TRP, humic acid + L‐tryptophan; HA + NaCl, humic acid + sodium chloride; HA, humic acid; K, potassium; LBL, leaf blade length; LBW, leaf blade width; LL, leaf length; L‐TRP + NaCl, L‐tryptophan + sodium chloride; L‐TRP, L‐tryptophan; MDA, malondialdehyde; Mg, magnesium; Mn, manganese; Na, sodium; NaCl, sodium chloride; Ni, nickel; NL, number of leaves; P, phosphorus; PDW, plant dry weight; PFW, plant fresh weight; PH, Plant height; POD, peroxidase; PPO, polyphenol oxidase; RL, root length; RWC, relative water content; S, sulfur; SOD, superoxide dismutase; Zn, zinc.

Two‐way clustered heatmap was drawn to observe the effect of HA and L‐TRP treatments on different traits of spinach plants depending on salt stress conditions (Figure 1). The examined parameters are grouped according to their similarity in specific treatments and the correlation is shown by colored squares. Blue color indicates positive correlation and green indicates negative correlation of parameters affected by treatments.

Analysis of the heatmap showed that the growth parameters were divided into four main groups. There are PH and LBL in the first group, PFW and LL in the second group, PDE and LBW in the third group, and RL and NL in the fourth group (Figure 1a). According to heatmap analysis, HA + L‐TRP treatment was found to have a significant positive effect on PH and PFW. On the other hand, it was determined that NaCl treatment had a negative effect on all growth parameters examined. HA treatment appeared to have no remarkable effect on growth parameters. It has been observed that HA + L‐TRP + NaCl treatment, which is applied to reduce the negative effects of salt, gives better results compared to HA + NaCl and L‐TRP + NaCl treatments. These findings are consistent with the data presented in Table 1.

When the heatmap is examined, mineral contents are grouped into two main groups. While Fe content constitutes the first group, all other mineral contents are collected in the second group. When the treatments were examined, salt‐containing treatments were in the first group (NaCl, HA + NaCl, L‐TRP + NaCl, and HA + L‐TRP + NaCl), while other treatments were in the second group (control, HA, L‐TRP, and HA + L‐TRP) (Figure 1b). In general, mineral contents showed a decrease, that is, a negative correlation, due to HA, L‐TRP, and HA + L‐TRP treatments. On the other hand, Fe content showed a positive correlation with these treatments.

When the heatmap of photosynthetic pigments, antioxidant compounds, oxidative stress, and antioxidant enzyme activities was examined, two main groups were formed. The first group included MDA, H_2_O_2_, and proline. The remaining contents are collected in four subclusters in the second group. The first cluster included chlorophyll a, SOD, GB, and POD, the second cluster included CAT, RWC, and polyphenol, the third group included anthocyanin, APX, lutein, and PPO, and the fourth group included chlorophyll b, total chlorophyll, and total carotenoid. When the heatmap is examined, the treatments are grouped into two groups. While the first group included HA, L‐TRP, and HA + L‐TRP, the second group included NaCl, control, HA + NaCl, L‐TRP + NaCl, and HA + L‐TRP + NaCl (Figure 1c). While a strong positive correlation emerged between NaCl salt stress and MDA and H_2_O_2_, a weak positive correlation emerged with proline. On the other hand, a strong negative correlation was detected between NaCl salt stress and all other contents. It was concluded that, compared to the control, the healing effect of HA + L‐TRP treatment on photosynthetic pigments, antioxidant compounds, oxidative stress, and antioxidant enzyme activities was higher than other treatments.

## 4 CONCLUSIONS

The current study demonstrated that salt, HA, and L‐TRP treatments significantly affected growth parameters and biochemical composition of spinach. Salinity stress negatively influenced plant growth, and parameters like PH, NL, leaf dimensions, PFW, and PDW significantly reduced depending on salt stress. Conversely, individual and combined applications of HA and L‐TRP markedly improved all growth parameters. Additionally, salinity caused a considerable decrease in chlorophyll, carotenoid, polyphenol, lutein, anthocyanin, PPO, GB, RWC, and antioxidant enzyme activities of spinach. However, significant increases were detected in Na, Cl, K, S, Zn, Ni, proline, MDA, and H_2_O_2_ contents of spinach due to salinity. Foliar applications of HA and L‐TRP, either separately or together, positively influenced plant growth, RWC, activities of antioxidant enzymes, chlorophyll, and essential mineral levels in spinach under both normal and saline conditions.

The results suggest that the combined use of HA and L‐TRP is a promising strategy for mitigating the negative impacts of salinity on spinach. This approach can enhance spinach cultivation in saline environments, contributing to agricultural sustainability and food security. Therefore, HA and L‐TRP foliar applications can be recommended as effective practices for improving spinach growth under salt stress, providing a potential alternative for cultivating this economically significant vegetable in challenging conditions.

## AUTHOR CONTRIBUTIONS

**Nezahat Turfan:** Data curation (equal); investigation (equal); project administration (equal); resources (equal); supervision (equal). **Beyhan Kibar:** Investigation (equal); methodology (equal); writing – original draft (equal). **Nazakat Davletova:** Data curation (equal); investigation (equal). **Hakan Kibar:** Investigation (equal); resources (equal); software (equal); supervision (equal); writing – review and editing (equal).

## FUNDING INFORMATION

This research was not funded.

## CONFLICT OF INTEREST STATEMENT

The authors declare that they do not have any conflicts of interest.

## CONCEPT FOR PUBLICATION

All authors have approved the article for publication.

## Data Availability

Data are available on request from the authors.
